# Modern cancer therapy: cryoablation meets immune checkpoint blockade

**DOI:** 10.3389/fonc.2024.1323070

**Published:** 2024-02-07

**Authors:** Qi Liu, Chunyang Zhang, Xuxin Chen, Zhihai Han

**Affiliations:** ^1^ Department of Pulmonary and Critical Care Medicine, The Sixth Medical Center of Chinese People’s Liberation Army (PLA) General Hospital, Beijing, China; ^2^ Navy Clinical College, the Fifth School of Clinical Medicine, Anhui Medical University, Hefei, Anhui, China; ^3^ College of Pulmonary and Critical Care Medicine, Chinese People’s Liberation Army (PLA) General Hospital, Beijing, China

**Keywords:** cryoablation, immune checkpoint blockade, immunotherapy, combined therapy, cancer immunology

## Abstract

Cryoablation, as a minimally invasive technology for the treatment of tumors, destroys target tumors with lethal low temperatures. It simultaneously releases a large number of tumor-specific antigens, pro-inflammatory cytokines, and nucleoproteins, known as “danger signals”, activating the body’s innate and adaptive immune responses. However, tumor cells can promote the inactivation of immune effector cells by reprogramming immune checkpoints, leading to the insufficiency of these antigens to induce an immune response capable of eradicating the tumor. Immune checkpoint blockers rejuvenate exhausted T cells by blocking immune checkpoints that induce programmed death of T cells, and are therefore considered a promising therapeutic strategy to enhance the immune effects of cryoablation. In this review, we provide a detailed explanation of the immunological mechanisms of cryoablation and articulate the theoretical basis and research progress of the treatment of cancer with cryoablation combined with immune checkpoint blockers. Preliminary data indicates that this combined treatment strategy exhibits good synergy and has been proven to be safe and effective.

## 1 Introduction

In this century, cancer is set to become the leading cause of premature death worldwide (1). Despite some significant achievements in the field of cancer treatment, overall cancer-related mortality remains relatively stable (2). Over the past few decades, the five-year survival rate for lung cancer, liver cancer, and esophageal cancer has hovered around 15%, and the five-year survival rate for cancer patients with distant metastases is less than 10% (3). Hence, in the face of these challenges, it is imperative to explore new treatment techniques and optimize treatment plans.

Currently, ablation techniques, as a minimally invasive treatment approach, have been widely implemented in clinical practice. They offer a safe and effective alternative for elderly patients who are not suitable for surgery, patients with underlying health conditions, and those at a higher surgical risk. Compared to thermal ablation methods like radiofrequency ablation, cryoablation demonstrates advantages in ease of operation, reduced patient pain, clear delineation of the ablation zone, and adaptability to tumor shapes through multi-needle combination. Therefore, in certain cases, it is considered a viable treatment option (4–9). In the proposed cryoimmunotherapy mechanism, cryoablation induces coagulative necrosis in tumor tissues through ultra-low temperature physical methods (10–12), while simultaneously releasing tumor-specific antigens *in situ* (akin to autologous vaccine inoculation), promoting the body’s anti-tumor specific immune response (13–15). However, numerous studies show that the immune activation capability of cryoablation alone is limited. Therefore, it is important to seek immunotherapy that can assist cryoablation in generating stronger immune effects.

In 2017, the immune checkpoint blockade (ICB) pembrolizumab (anti-PD-1) was added to the first-line anti-tumor therapy, significantly improving the overall survival (OS) of patients (16, 17). ICBs enhance the body’s anti-tumor immune response by blocking immune checkpoints that cause programmed death of T cells, thus preventing T cell exhaustion (18–21). However, a drawback of immunotherapy is the low objective remission rate (22–24). In addition, certain genetic mutations can lead to primary resistance to ICBs, and some patients may develop acquired resistance or disease hyper-progression (25–31). Therefore, it is important to find combination therapies that can expand the ICB beneficiary population and enhance its immunotherapeutic effects.

Here, we elucidate the immunological mechanisms of cryoablation. Concurrently, we summarize the clinical and preclinical research on cryoablation combined with ICBs in tumors and detail the theoretical basis and potential advantages supporting this combination approach. Finally, we discuss the challenges faced by this combined approach and its future directions.

## 2 Cryoablation

**Figure 1:**
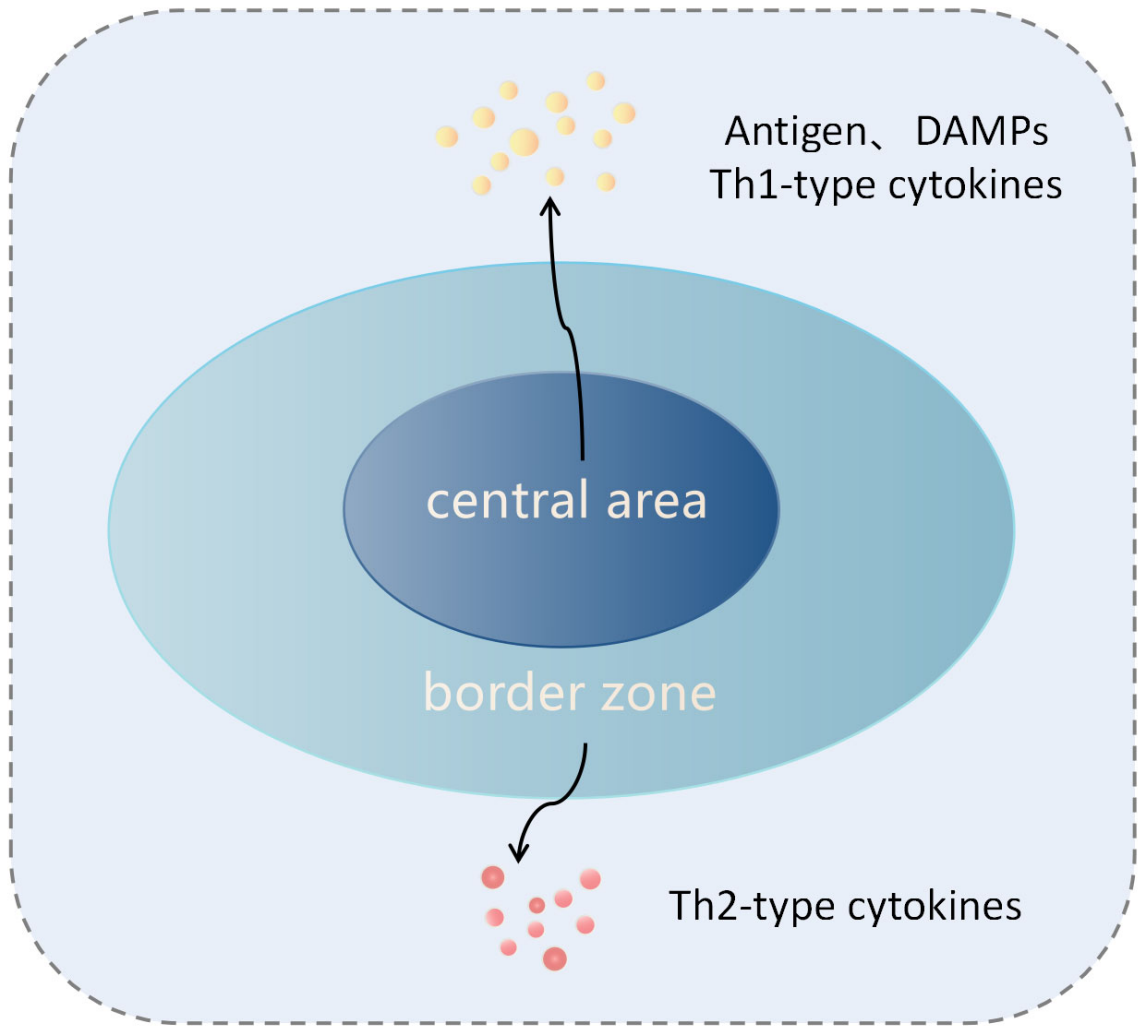
lustration of tumor tissue after cryoablation. The cryoablation zone is divided into the central zone and the edge zone. In the central zone, tumor cells undergo coagulative necrosis and release antigens and damage-associated molecular patterns (DAMPs), which induce innate and adaptive immune responses against the tumor. In the sublethal temperature zone at the edge of the ablation, tumor cells undergo apoptosis, and this region is characterized by a Th2 cytokine environment, inducing immune tolerance.

The cryoablation technique utilizes the Joule-Thomson effect by releasing compressed liquid gas into the target tumor tissue via specialized cryoablation probes. As the compressed liquid gas rapidly expands and transforms into a gaseous state, ultra-low temperatures are produced, causing the temperature of the target tissue to rapidly drop to around -140°C. Following cryoablation, the ablation zone can be divided into a central region and a peripheral region (**Figure 1**). In the central area, which is closer to the probe, intracellular fluid forms ice crystals, leading to physical cellular damage; the extracellular fluid freezes, resulting in osmotic cellular damage. Both types of injuries ultimately lead to coagulative necrosis of the tumor cells (32, 33). However, in the peripheral region where lethal temperatures are not reached, damaged cells that have not undergone necrosis mediate tumor cell apoptosis through the mitochondrial pathway (32).

**Figure 2:**
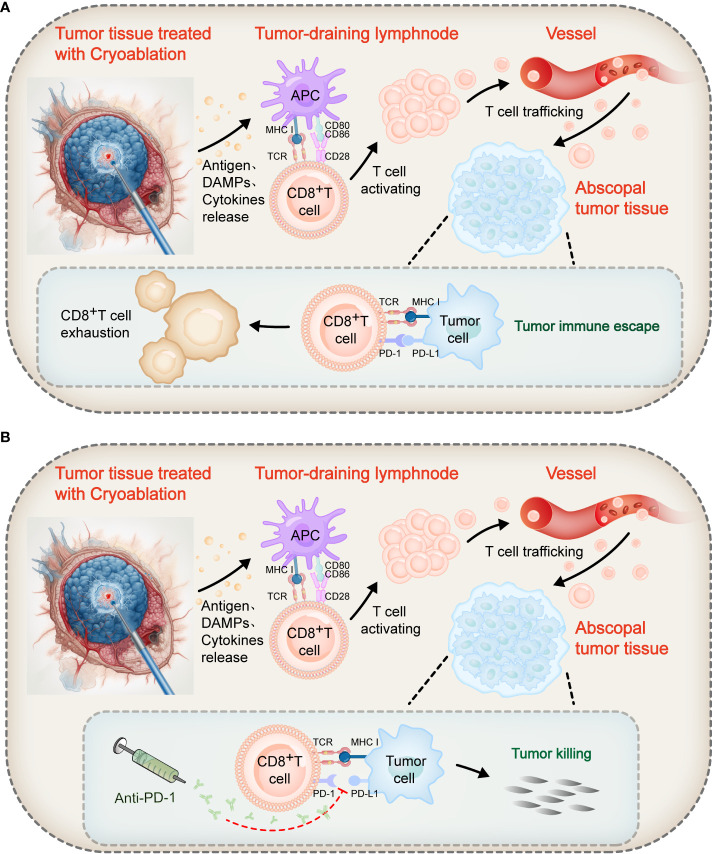
Mechanism of action of cryoablation combined with immune checkpoint blockers. Following cryoablation, coagulative necrotic tumor tissue releases a large number of antigens, prompting APCs to uptake these antigens and increase the expression of MHC molecules and co-stimulatory ligands CD80/CD86. T lymphocyte activation requires two signals: Firstly, the TCR recognizes antigen peptides bound to MHC molecules on APCs, providing an antigen recognition signal; secondly, the co-stimulatory receptor CD28 on T cells binds with CD80/CD86 on APCs, transmitting a co-stimulatory signal. These two signals together promote the activation and proliferation of T cells. Activated tumor-specific T cells can recognize and destroy both local and distant tumor cells, realizing the immunological effect of cryoablation. **(A)** However, in the abscopal tumor tissue, the binding of PD-1 on some T cell surfaces to PD-L1 on tumor cells inhibits co-stimulatory signals, leading to impaired proliferation and reduced cytotoxicity of T cells, transforming them into “exhausted” T cells, and thus triggering tumor immune escape. **(B)** The combined use of PD-1 inhibitors can block the PD-1/PD-L1 axis, restoring the signaling pathway and thus reviving the proliferative and effector capabilities of these “exhausted” T cells to achieve the objective of killing tumor cells.

In the central region, necrotic tumor cells release antigens (such as cell surface antigens, intracellular, and nuclear antigens) as well as damage-associated molecular patterns (DAMPs: endogenous molecules released from dying cells, such as high mobility group box 1 (HMGB1), DNA, calreticulin, or f-actin) (11, 15). Among these, DAMPs are believed to activate dendritic cells (DCs) through Toll-like receptors (TLRs) such as TLR4, promoting the generation of a specific immune response against tumor antigens (34, 35). Furthermore, some DAMPs, such as pro-inflammatory cytokines and nuclear proteins, can attract and activate neutrophils, macrophages, and natural killer (NK) cells, stimulating an innate immune response. Activated NK cells can directly lyse tumor cells (32, 36). Additionally, research on autoimmune diseases has shown that nuclear and organelle-derived antigens may be more effective stimulants for the host’s innate and adaptive immune systems (37). Under the influence of cytokines and chemokines in the central region of cryoablation, immature dendritic cells (IDC) reach the damaged tissue, and in the context of inflammation and abundant cytokines, take up the above tumor antigens (13). IDC enters the draining lymph nodes through the afferent lymphatic vessels, where they differentiate into mature DC, upregulating the expression of the Major Histocompatibility Complex (MHC) molecules, co-stimulatory molecules, and adhesion molecules. They then present these tumor-specific antigens through MHC class I and II molecules, activating CD8^+^T and CD4^+^T cells (13, 32). Moreover, within the activated CD4^+^ T lymphocytes, the helper T cell 1 (Th1) subset can release various Th1 cytokines, such as interleukin-2 (IL-2), interferon-γ (IFN-γ), and tumor necrosis factor-alpha (TNF-α). These cytokines promote the proliferation of CD8^+^ T cells and their differentiation into cytotoxic T lymphocytes (CTL), enhancing the anti-tumor immune response (32) (**Figure 2A**). These activated tumor-specific T cells possess the ability to recognize and destroy both local and distant tumor cells, thereby killing tumor cells and inhibiting the growth of untreated tumors at distant sites. This demonstrates the abscopal effect of cryoablation. However, unlike necrotic cells, apoptotic cells in the marginal area not only fail to stimulate an immune response but can also induce immune tolerance (32, 38). In the marginal region, after phagocytizing apoptotic cells, macrophages and dendritic cells do not upregulate the expression of co-stimulatory molecules. Additionally, they secrete anti-inflammatory cytokines such as transforming growth factor-β (TGF-β1) and interleukin-10 (IL-10), leading to the inability to activate T cells (35, 39) (**Figure 1**). Current research suggests that the ratio of immunogenic necrosis induced by cryoablation to immune tolerogenic cell apoptosis is a decisive factor leading to anti-tumor immune responses or immune tolerance. Therefore, we should continue to improve cryoablation techniques, increase the area of tumor cell necrosis, reduce the area of apoptosis, and find the best cryoablation conditions to induce anti-tumor immune responses.

A vast body of literature indicates that cryoablation has immunological effects. Cryoablation not only directly destroys tumor cells at the local treatment site but also activates the body’s immune system, impacting metastatic tumors at distant sites that have not been treated – a phenomenon known as the abscopal effect. A study by Sabel et al. (40, 41) found that in mice with cryoablated or surgically removed breast cancer, the recurrence rate in the cryoablation group was significantly lower than in the surgical group (16% vs 86%). After cryoablation, levels of interleukin-12 (IL-12) and IFN-γ in the mice’s serum significantly increased, and T-cell toxicity in tumor-draining lymph nodes (TDLNs) was significantly enhanced. Transferring cryoablated TDLNs to untreated tumor-bearing mice resulted in fewer lung metastases and a higher proportion of tumor-specific T cells, compared to the group receiving TDLNs from surgically removed tumors. These results suggest that cryoablation can trigger a tumor-specific T cell response in TDLNs, inhibiting the growth of secondary tumors. A study by Den Brok et al. (42) found that when necrotic tumor tissue was left *in situ* after cryoablation, 50% of B16-OVA tumor-bearing mice could resist re-challenge experiments with B16-OVA cells. In contrast, when necrotic tumor tissue was removed immediately after cryoablation, the survival rate of the mice subjected to subsequent challenge experiments dropped to 0%. This suggests that the anti-tumor immune response induced by cryoablation heavily relies on the necrotic tumor tissue produced by the cryoablation process. It further suggests that cryoablation of solid tumors can release tumor antigens in situ, thereby creating an antigen reservoir essential for inducing *in situ* an anti-tumor immune response. Takahashi Y et al. (43) investigated the impact of one, two, and three cycles of cryoablation on the immune modulation of abscopal tumors by inoculating Lewis lung carcinoma and B16 melanoma cells in bilateral flanks of mice. The results showed that mice undergoing two freeze/thaw cycles on the left-sided tumor had the longest survival, the slowest growth rate of the right-sided tumor, and a higher proportion of CD4^+^ and CD8^+^ tumor-infiltrating lymphocytes(TILs). Additionally, there was a significant increase in the levels of IL-1β, IL-2, IL-6, IL-12β, IFN-γ, and TNF-α in the lavage fluid surrounding the cryoablated tumor. This suggests that two cycles of cryoablation effectively activate pro-inflammatory cytokines, enhancing the activity of immune cells in abscopal tumors, thereby producing the strongest abscopal effect. In a study conducted on a mouse model of prostate cancer (44), compared with the surgical and control groups, mice treated with cryoablation exhibited suppressed growth of distant untreated tumors, reduced rates of lung metastasis, extended survival, and an increase in the proportion and activity of CD4^+^ and CD8^+^ T cells as well as NK cells in peripheral blood. This indicates that the abscopal effect generated by cryoablation is mediated by an anti-tumor immune response. In a study on a breast cancer tumor-bearing mouse model, Rakhshanda L. Rahman et al. (45) found that compared to the baseline group, mice treated with cryoablation showed a significant increase in TILs in both the stroma and margins of distant tumors (2.8% increase in stroma, p = 0.015; 50% increase at the margin, p = 0.02), as well as a significant rise in CD8^+^ T cells and granzyme B (GzmB) (increases of 15.7, p = 0.02, and 4.8, p = 0.048, respectively). The cryoablation group of mice exhibited no recurrence or metastasis, whereas 40% of the mice in the surgical group showed recurrence and lung metastasis. Proportional tests further revealed a significant correlation between the increase in the percentage of TILs in distant tumors and the prevention of tumor recurrence (p = 0.02). These results suggest that cryoablation, compared to traditional surgical removal, more effectively stimulates TIL responses and prevents cancer recurrence and metastasis through the abscopal effect. In another study on a breast cancer tumor-bearing mouse model, Wenjun Fan et al. (46) observed that mice treated with cryoablation had prolonged survival and an increase in the number of CD8^+^T and CD4^+^T cells in distant tumors compared to the untreated control group. Further multi-omics analysis revealed that cryoablation activates the lysosomal pathway in tumor tissues, leading to overexpression of key proteins such as SNAP23 (Synaptosome Associated Protein 23) and STXBP2 (Syntaxin Binding Protein 2). This process not only promotes the activation of immune effector cells but also suppresses immunosuppressive cells like regulatory T cells (Treg) and M2 macrophages. The study confirms that cryoablation enhances anti-tumor immune responses and induces the abscopal effect, effectively prolonging the survival of mice. Clinical trials have also obtained similar results. Osada et al. (47) found that among patients with liver metastatic tumors who underwent cryoablation treatment, those who exhibited an “abscopal effect” showed a significantly higher Th1/Th2 ratio in their peripheral blood compared to those who did not experience this effect. This observation suggests that the enhanced systemic anti-tumor immune response following cryoablation can mediate the abscopal effect in patients. In another study on cryoablation in 22 renal cancer patients (15), the authors, through T cell receptor β (TCRβ) sequencing and TCR diversity analysis of tissue and peripheral blood samples before and after cryoablation, found an increase in anti-tumor-specific T cells in both local tumor tissues and peripheral blood of renal cancer patients who underwent cryoablation. Concurrently, the study also observed an increase in the number of tumor-infiltrating DCs. This experiment indicates that cryoablation induced both local and systemic anti-tumor immune responses in renal cancer patients. In summary, the aforementioned data provides substantive immunological evidence for the cryoablation therapy, demonstrating that cryoablation can stimulate a tumor-specific immune response and effectively promote the occurrence of the abscopal effect.

Despite these findings, some experiments have shown that cryoablation does not inhibit tumor growth (48), and some results even suggest that cryoablation may increase the rate of metastasis and mortality in Tumor transplant mouse model (such as the fibrosarcoma KMT-17 transplant rat model) (49). Current research suggests that the conditions of cryoablation are closely related to the intensity of the anti-tumor immune response, and these inconsistent experimental results may be related to factors such as the temperature of cryoablation, freezing time, freeze-thaw cycle number, and ablation area.

Furthermore, unlike the denatured antigens released by thermotherapy-based ablation (such as radiofrequency ablation, microwave ablation, high-intensity focused ultrasound, etc.), cryoablation releases natural tumor antigens that retain their full immunogenicity by destroying tumor tissue through ultra-low temperatures. This feature allows cryoablation to induce a stronger immune response than thermal ablation (50). In the study by Shao et al. (50), they utilized the B16-F10 cell line (B16), which is a mouse melanoma cell line, to assess the ability of cryoablation and thermal therapy to release proteins, antigens, and activate tumor antigen-specific CD8^+^T cells. The results indicated that after cryoablation treatment of B16 tumors, the amount of protein released and the known antigen, Tyrosinase-related protein 2 (TRP-2), were both significantly higher than that with thermal therapy. Moreover, unlike thermal therapy, which released a substantial amount of denatured proteins (72.9 ± 18.1%), the proteins released by cryoablation were almost all in their native form, with denatured proteins accounting for only (7.35 ± 28.2%). This study elucidated, at the cellular level, the mechanism by which cryoablation is superior to thermal therapy in promoting T cell activation and proliferation. Den Brok et al. (13) also found that in TDLNs, the proportions of antigen-bearing DCs and mature DCs in the cryoablation group were higher than in the radiofrequency ablation group. Also, when combined with ICB treatment, the cryoablation combination group was stronger than the radiofrequency ablation combination group in inducing the quantity and function of tumor-specific T cells. These results suggest that cryoablation, compared to other thermal ablation techniques, may be more suitable for combination with immunotherapy to enhance the body’s anti-tumor immune response.

## 3 Immune checkpoint blockade

In the past decade, ICB has made significant progress in the treatment of advanced malignancies. It employs monoclonal antibodies to inhibit immune checkpoint molecules, activating T lymphocytes, including tumor-specific T cells, thereby enhancing the immune system’s ability to attack tumor cells. However, due to the excessive activation of T cells, the body’s self-tolerance is lost, leading to immune-related adverse events in some patients, such as skin inflammation, gastrointestinal issues, and liver function abnormalities (18, 51).

The immune checkpoints currently targeted by immunotherapy mainly include programmed cell death protein 1 (PD-1, CD279), programmed cell death ligand 1 (PD-L1), and cytotoxic T lymphocyte-associated antigen-4 (CTLA-4).

### 3.1 CTLA-4 blockers

CTLA-4 is an inhibitory receptor specifically expressed on the surface of T cells. Its expression is upregulated upon T cell activation, thereby preventing sustained T cell activation in the early stages of an immune response (52–54). Once the TCR signal is activated, CTLA-4 repositions from within the cell to the cell surface and competitively binds to the CD80/CD86 molecules on the surface of Antigen-Presenting Cells (APCs), in competition with the T cell’s CD28. It’s important to note that CTLA-4 has 20 times the affinity for CD80/CD86 than CD28 does, which results in the suppression of co-stimulatory signal transmission, thus exerting an immune inhibitory effect (55). CTLA-4 blockers can lift the restrictions CTLA-4 places on T cell signaling, allowing antitumor lymphocytes to continue their effector responses against tumor cells (56).

Ipilimumab and Tremelimumab are drugs that can block the CTLA-4 checkpoint. Both of these drugs have been proven to enhance the body’s anti-tumor immune response. In 2011, The U.S. Food and Drug Administration (FDA) has approved ipilimumab for the treatment of patients with advanced melanoma. In 2020, FDA has approved the combination of Ipilimumab and Nivolumab, along with two cycles of platinum-based dual chemotherapy, as a first-line treatment for patients with metastatic or recurrent non-small cell lung cancer (NSCLC) that do not have epidermal growth factor receptor (EGFR) or anaplastic lymphoma kinase (ALK) genomic tumor aberrations (57). Currently, research on Tremelimumab is ongoing in phase III clinical trials for urothelial carcinoma, non-small cell lung cancer, small cell lung cancer, and head and neck squamous cell carcinoma, as well as in phase I/II clinical trials for hepatocellular carcinoma (58–64).

### 3.2 PD-1/PD-L1 inhibitor therapy

PD-1 is also a negative co-stimulatory molecule, expressed on the surface of T lymphocytes, B cells, macrophages, and other cells, and is a member of the B7-CD28 family. PD-1 has two ligands, namely PD-L1 (also known as B7-H1 or CD274) and PD-L2 (B7-DC, CD273). In tissues, the expression of PD-L1 and PD-L2 can inhibit local immune responses, control tissue damage, and maintain immune tolerance in tissues (65). However, tumor cells use PD-L1 as a molecular “shield”. When PD-L1 binds to PD-1, the T cells’ ability to proliferate decreases and their cytotoxic ability diminishes, transforming into “exhausted” T cells, leading to immune evasion of the tumor (66–74) (as shown in **Figure 2A**). However, PD-1/PD-L1 blockers can block the PD-1/PD-L1 axis, thereby reactivating these exhausted T cells, restoring their proliferation and effector capabilities, and thereby killing tumor cells (74, 75) (as shown in **Figure 2B**). Based on this mechanism, PD-1/PD-L1 blockers have become a valuable method of enhancing the killing power of T lymphocytes in anti-tumor immunotherapy.

Currently, the FDA has approved five anti-PD-1 or anti-PD-L1 antibodies (pembrolizumab, nivolumab, atezolizumab, durvalumab, and avelumab) for the treatment of 11 types of cancer, including refractory melanoma, advanced non-small cell lung cancer, Merkel cell carcinoma, Hodgkin’s lymphoma, highly microsatellite instability (MSI-h) tumors, head and neck cancer, bladder cancer, urothelial carcinoma, gastric esophageal cancer, hepatocellular carcinoma, and renal cell carcinoma (69).

However, it is worth noting that not all types of cancer respond to ICB. In fact, only about 20-30% of patients in cancer types that have shown response to ICB can benefit from this treatment. Additionally, some patients may experience acquired resistance or disease progression (76–80). Therefore, it is crucial to search for combination therapies that can broaden the population of beneficiaries from ICB immunotherapy and enhance immune efficacy.

## 4 Combination of cryoablation and immune checkpoint blockade

### 4.1 Theoretical basis for combining cryoablation with immune checkpoint blockers

Due to the heterogeneity of malignant tumors and the complexity of immune regulatory mechanisms, monotherapy often has limitations, and therefore, combination therapy is becoming a major strategy for clinical cancer treatment (81). When considering rational combination therapies, the primary focus is on how to combine drugs to alleviate tumor burden and enhance anti-tumor immune responses, thus improving patient prognosis (81). Numerous studies have indicated that cryoablation not only reduces tumor burden in the local treatment area through physical means but also activates the immune system to recognize and attack distant tumor cells by releasing tumor antigens, thereby inducing the abscopal effect (11, 13–15). The addition of ICBs further enhances this systemic immune response by blocking inhibitory signals, thus strengthening the ability of T cells to attack tumor cells. Furthermore, it is recognized that the quantity of pre-existing T cells in the tumor microenvironment is intimately correlated with the therapeutic efficacy of ICB (82–84). Therefore, combining ICB with cryoablation could significantly enhance the efficacy of each treatment modality when used alone (85).

It is well-known that the activation of naïve T lymphocytes requires dual signals: The first signal involves the recognition of antigenic peptides bound to the MHC molecules on the surface of APCs through the TCR, thereby transmitting an antigen recognition signal. The second signal comes from the binding of the co-stimulatory receptor CD28 on the T cell surface to the co-stimulatory ligands CD80/CD86 on the APC surface, thereby transmitting a co-stimulatory signal. With the combined stimulation of the first and second signals, T cell activation and proliferation are induced (38, 86) (**Figure 2A**). Cryoablation can release of tumor antigens, inducing APCs to uptake antigens and upregulate the expression of MHC molecules and CD80/CD86, thereby promoting T lymphocyte activation (13, 32, 83). However, in tumor tissues, PD-1 and PD-L1 are often overexpressed. The PD-1/PD-L1 axis can block the transmission of the second signal, also known as the co-stimulatory signal, resulting in T lymphocytes becoming unresponsive (87) (**Figure 2A**). Similarly, CTLA-4 binding to CD80/CD86 on APCs also prevents co-stimulatory signal transmission, exerting an immune inhibitory effect (55, 88). Tumors exploit these immune checkpoints as escape mechanisms, blocking further activation and proliferation of T cells. As a result, tumor antigens released by cryoablation are insufficient to induce a significant anti-tumor immune response (38, 89). However, PD-1/PD-L1 blockers can block the binding of PD-1 to PD-L1, while CTLA-4 blockers can disrupt the binding of CTLA-4 to CD80/CD86. This releases the “braking” mechanism of the co-stimulatory signal pathway, restores signal transmission, and consequently revives the activation and proliferation of T cells (56, 74, 75, 90). (**Figure 2B**). These tumor-specific T cells, synergistically activated by cryoablation and ICB treatment, possess the capability to recognize and destroy both local residual tumors and distant macroscopic and microscopic metastases. The aforementioned mechanism provides a theoretical foundation for the combined application of cryoablation and immunotherapy.

### 4.2 Research progress on the combination of cryoablation and immune checkpoint blockers

Here, we summarize the research progress on the combination of cryoablation and ICB therapy in treating specific types of tumors. These studies include preclinical mouse model research and preliminary results from clinical trials (**Tables 1**, **2**). Our analysis indicates that this combined therapy exhibits a notable synergistic effect in enhancing anti-tumor immune responses. Furthermore, a series of related clinical trials are currently underway (**Table 3**), and the progression of these trials will provide further evidence for assessing the effectiveness of this combination therapy in clinical applications.

**Table 1:** Pre-clinical mouse studies on the combination of cryoablation and ICB therapy.

Study (year)	Tumor model	Treatment	Significant findings	Ref.
Waitz R et al.(2012)	Prostate cancer TRAMP C2 in C57BL/6N mice	Cryo and Anti-CTLA Ab	Enhanced antitumor immune responses and metastatic tumor rejection reactions	(91)
Benzon B et al.(2018)	Prostate cancer TRAMP C2 in C57BL/6N mice	Cryo and Anti-CTLA Ab	Delayed tumor growth, reduced mortality in mice, and enhanced antitumor immune responses	(92)
den Brok et al.(2006)	Melanoma B16F10 in C57BL/6N mice	Cryo and Anti-CTLA Ab	Enhanced antitumor immune responses and protected mice from secondary tumor challenges	(13)
Benzon B et al.(2018)	Renal cell carcinoma RENCA in BALB/C mice	Cryo and Anti-PD-1 Ab	Distant tumor growth was inhibited, and the body’s antitumor immune response was enhanced	(93)
Jin Y et al.(2023)	BC 4T1 in BALB/C mice	Cryo and Anti-PD-1 Ab	Reduced the tumor’s immunosuppression and amplified the Cryo-triggered immune response	(94)

Cryo, Cryoablation; Ab, Antibody.

**Table 2:** Clinical trials on the combination of cryoablation and ICB therapy.

Study (year)	Patient population	Treatment	Significant findings	Ref.
McArthur HL et al.(2016)	Female patients with breast cancer	Cryo and Ipilimumab	Local and systemic antitumor immune responses were enhanced, and the treatment was safe with good tolerability.	(95)
Campbell MT et al.(2021)	Patients with metastatic renal cell carcinoma	Cryo and Tremelimumab	Improved the immunosuppressive microenvironment and increased tumor-infiltrating T lymphocytes	(96)
Kim DW et al. (2015)	Patients with metastatic melanoma	Cryo, Ipilimumab or Pembrolizumab	Improved survival rate and disease control rate, with safety and good tolerability	(97)
Shen L et al.(2020)	Patients with melanoma liver metastases	Cryo and Pembrolizumab	Enhanced antitumor immune responses, with safety and good tolerability	(98)
Feng J et al.(2021)	Patients with advanced NSCLC	Cryo and Nivolumab	Cryoablation combined with nivolumab is safe and well-tolerated, and it outperforms cryoablation alone in improving the clinical efficacy in patients with advanced NSCLC	(99)

Cryo, Cryoablation.

**Table 3:** Cryoablation combined with immune checkpoint blockers.

NCT ID	Title	Type of immune therapy combined with cryoablation	Phase
NCT03546686	Peri-Operative Immune Checkpoint Inhibition and Cryoablation in Women With Triple-negative Breast Cancer	Pembrolizumab	Phase II
NCT05806385	Grouping Immune-modulation With Cryoablation (LOGIC) for Breast Cancers (LOGIC)	Pembrolizumab	Phase I Phase II
NCT01502592	Pre-Operative, Single-Dose Ipilimumab and/​or Cryoablation in Early Stage/​Resectable Breast Cancer	Ipilimumab	Phase I
NCT02833233	A Study of Pre-Operative Treatment With Cryoablation and Immune Therapy in Early Stage Breast Cancer	Ipilimumab and Nivolumab	Not Applicable
NCT04249167	Cryoablation, Atezolizumab/​Nab-paclitaxel for Locally Advanced or Metastatic Triple Negative Breast Cancer	Atezolizumab and Nab-paclitaxel	Early Phase I
NCT05781074	Cryoablation Combined With Sintilimab Plus Lenvatinib in Patients With Immune Checkpoint Inhibitor Previously Treated Advanced Biliary Tract Cancer (CASTLE-08) (CASTLE-08)	Sintilimab and Lenvatinib	Phase II
NCT02821754	A Pilot Study of Combined Immune Checkpoint Inhibition in Combination With Ablative Therapies in Subjects With Hepatocellular Carcinoma (HCC) or Biliary Tract Carcinomas (BTC)	Durvalumab and Tremelimumab	Phase II
NCT03290677	Study of Core Needle Biopsy and Cryoablation of an Enlarging Tumor in Patients With Metastatic Lung Cancer and Metastatic Melanoma Receiving Post-progression Immune Checkpoint Inhibitor Therapy	Immune checkpoint inhibitor	Not Applicable
NCT04339218	Cryoablation in Combination (or Not) With Pembrolizumab and Pemetrexed-carboplatin in 1st-line Treatment for Patients With Metastatic Lung Adenocarcinoma (CRYOMUNE)	Pembrolizumab and Pemetrexed-carboplatin	Phase III
NCT02469701	Advanced Non-Small Cell Lung Cancer Progressing After at Least One Prior Therapy For Metastatic Disease	Nivolumab	Phase II
NCT05779423	Cryoablation+Ipilimumab+Nivolumab in Melanoma	Ipilimumab and Nivolumab	Phase II
NCT03325101	Dendritic Cell Therapy After Cryosurgery in Combination With Pembrolizumab in Treating Patients With Stage III-IV Melanoma That Cannot Be Remove by Surgery	Pembrolizumab and Therapeutic Autologous Dendritic Cells	Phase I Phase II
NCT04701918	Pembrolizumab And Cryoablation In Urothelial Carcinoma	Pembrolizumab	Phase II
NCT02423928	Phase I Clinical Trial of Cryoimmunotherapy Against Prostate Cancer (CryoIT)	Cyclophosphamide, Ipilimumab, and Autologous Immature Dendritic Cells Therapy	Phase I
NCT04090775	A Phase 2 Trial for Men With Metastatic Prostatic Adenocarcinoma	Nivolumab, Ipilimumab, and Cyclophosphamide	Phase II
NCT02489357	Pembrolizumab and Cryosurgery in Treating Patients With Newly Diagnosed, Oligo-metastatic Prostate Cancer	Pembrolizumab and Degarelix	Not Applicable
NCT03189186	Phase-I Trial of Pembrolizumab and Percutaneous Cryoablation Combination Followed by Nephron-Sparing Surgery or Cytoreductive Nephrectomy in Locally Advanced and Metastatic Renal Cell Carcinomas	Pembrolizumab	Phase I
NCT02626130	Tremelimumab With or Without Cryoablation in Treating Patients With Metastatic Kidney Cancer	Tremelimumab	Early Phase I
NCT03035331	Dendritic Cell Therapy, Cryosurgery, and Pembrolizumab in Treating Patients With Non-Hodgkin Lymphoma	Pembrolizumab and Dendritic Cell Therapy	Phase I Phase II
NCT04713371	A Phase 2 Trial for Patients With Metastatic Solid Cancer	Pembrolizumab, Ipilimumab, Cyclophosphamide, and GM-CSF Injectable	Phase II
NCT05302921	Neoadjuvant Dual Checkpoint Inhibition and Cryoablation in Relapsed/​Refractory Pediatric Solid Tumors	Nivolumab and Ipilimumab	Phase II
NCT04118166	Ipilimumab + Nivolumab + Cryotherapy in Metastatic or Locally Advanced Soft Tissue Sarcoma	Ipilimumab and Nivolumab	Phase II

#### 4.2.1 Pre-clinical mouse studies on the combination of cryoablation and ICB therapy

In this section, we will delve into the current basic research findings for several types of cancers, including prostate cancer, melanoma, renal cell carcinoma, and breast cancer. These studies offer preliminary insights into the potential of the combined therapy in cancer treatment.

In their research on prostate cancer TRAMP C2 bilateral tumor-bearing mice, Waitz R et al. (91) found that the combination of cryoablation with CTLA-4 inhibitors was more effective in inhibiting secondary tumor growth compared to monotherapy, and significantly increased the number of CD4^+^T and CD8^+^T cells in the tumor, as well as the ratio of effector T cells to Treg. These results suggest that the combined therapy can enhance anti-tumor immune responses and resist tumor metastasis. Further supporting these findings, Benzon B et al. (92) confirmed the advantages of this combined treatment in suppressing distant prostate cancer tumor growth and reducing mortality rates. Their research underscored the importance of T cells in the combined treatment, noting that the advantages of the combination therapy would be lost if T cells were depleted prior to treatment.

In their study of B16-OVA tumor-bearing mice, den Brok MH et al. (13) discovered that CTLA-4 inhibitors could enhance the weak anti-tumor immune response produced by cryoablation alone. The combined therapy not only protected the mice from secondary tumor attacks but also increased the number of tumor-specific T cells in the body and enhanced the secretion of IFN-γ.

Research on renal cell carcinoma mouse models (93) also supports these findings, showing that combined therapy (cryoablation and anti-PD-1 treatment) significantly inhibited the growth of distant tumors in mice. Immunological analysis revealed that in the tumors of mice receiving combined therapy, there was a significant increase in the infiltration of CD8^+^T lymphocytes and levels of IFN-γ and GzmB mRNA, with a significant decrease in IL-10 mRNA levels. This suggests that cryoablation combined with anti-PD-1 treatment is more effective in enhancing the body’s anti-tumor immune response compared to using either therapy alone.

In a recent study, Jin Y et al. (94), found in a breast cancer mouse model that cryoablation caused transient growth inhibition and an anti-tumor immune response in distant tumors, accompanied by an increase in PD-1/PD-L1 expression levels. When cryoablation was combined with an anti-PD-1 antibody, compared to monotherapy, there was a significant extension in the survival of mice, marked inhibition of distant tumor growth, and a significant increase in TILs. Additionally, the expression levels of anti-tumor immune cytokines such as TNF-α, IL-12α, T-bet, and GzmB mRNA were also elevated. These findings reveal that cryoablation combined with a PD-1 antibody can improve the immunosuppressive state of tumors and enhance the immune response induced by cryoablation, synergistically producing a more potent abscopal effect.

In summary, cryoablation combined with ICB therapy has shown significant anti-tumor potential in several types of cancers, including prostate cancer, melanoma, renal cell carcinoma, and breast cancer. Current research primarily focuses on describing changes in immune phenotypes, yet there remains a gap in deeply understanding the underlying mechanisms. This highlights the direction for future basic research.

#### 4.2.2 Clinical trials on the combination of cryoablation and ICB therapy

In this section, we will summarize key clinical trials to further evaluate the efficacy and potential value of cryoablation combined with ICB in patient treatment. In the clinical setting, this combined therapeutic approach has already begun to demonstrate its therapeutic potential. The following part will provide a comprehensive analysis of the results from several pivotal clinical studies:

In a preliminary clinical study (95), 19 female patients with breast cancer underwent preoperative tumor cryoablation, monotherapy with Ipilimumab, or a combination of both treatments, followed by breast surgery. The results showed that compared to the groups receiving cryoablation alone or Ipilimumab monotherapy, the combination therapy group had sustained elevation of peripheral blood levels of IL-2, IL-12, and IFN-γ. Activation and proliferation of CD4^+^ T and CD8^+^ T cells in peripheral blood and within the tumor also significantly increased. Among them, only one patient who underwent the combined treatment experienced a Grade 3 maculopapular rash, which is speculated to be related to the administration of the antiseptic chlorhexidine and/or cefamandole. This study suggests that the combination of cryoablation and Ipilimumab as a neoadjuvant therapy is safe, well-tolerated, and can synergistically induce local and systemic anti-tumor immune responses.

In another preliminary study on metastatic renal cell carcinoma (96), 18 patients with clear cell carcinoma and 11 patients with non-clear cell carcinoma underwent treatment with anti-CTLA-4 (Tremelimumab) monotherapy (n=14) or a combination of cryoablation and anti-CTLA-4 (n=15). The results showed a significant increase in tumor-infiltrating T lymphocytes in patients with clear cell carcinoma in the combination therapy group compared to anti-CTLA-4 monotherapy, while no similar phenomenon was observed in patients with non-clear cell carcinoma. These findings suggest that the combination of anti-CTLA-4 and cryoablation is feasible and can modulate the immune microenvironment in patients with metastatic clear cell renal carcinoma.

In a preliminary study (97) involving 16 metastatic melanoma patients who received a combination treatment of cryoablation and ICB (ipilimumab n=8 or pembrolizumab n=4) (97), the results showed a 6-month progression-free survival rate of 57%, a local disease control rate (DCR) of 83%, a distant DCR of 60%, and an overall DCR of 75%. This study demonstrates that the combination of cryoablation and ICB is safe, well-tolerated, and may be an effective strategy to enhance anti-tumor immune responses.

In another prospective cohort study (98) involving 15 patients with hepatic metastases from melanoma, it was shown that after a single cycle of cryoablation combined with a PD-1 blocker (pembrolizumab), there was a significant increase in NK cells in the patients’ peripheral blood and a decrease in Tregs. Furthermore, among these 15 patients, no grade 3-4 adverse events or major complications were observed. Of them, one case (7.3%) achieved complete remission, and three cases (20%) achieved partial remission. Numerous studies have reported that an increase in NK cells (100, 101) and a decrease in Tregs (102, 103) are associated with favorable immune responses in melanoma immunotherapy. These results indicate that the combination treatment is safe and effective, and can enhance anti-tumor immune responses in patients with melanoma liver metastases.

In a retrospective analysis of 64 patients with advanced NSCLC (99), patients who received cryoablation in combination with anti-PD-1(nivolumab) therapy (n=32) showed significant improvement in immune function and short-term efficacy (P < 0.05). Additionally, this group of patients had significantly lower levels of circulating tumor cells and tumor markers CYFRA21-1 and NSE compared to patients who received cryoablation alone (P < 0.05, n=32). Additionally, all adverse reactions were manageable. This study suggests that the combination of cryoablation and anti-PD-1 therapy has good tolerability, safety, and superior clinical efficacy compared to cryoablation alone in improving outcomes for patients with advanced NSCLC.

In a case report (104), a patient with metastatic renal cell carcinoma underwent CT-guided percutaneous cryoablation combined with local administration of anti-PD-1 (nivolumab). A follow-up PET scan one month after the procedure showed reduced uptake in two smaller metastatic bone lesions, with the smallest lesion completely eliminated. The largest bone metastasis showed slight shrinkage and increased uptake. The patient reported a significant reduction in hip pain and regained the ability to walk independently without assistance. This case report suggests that cryoablation combined with local administration of PD-1 blockers can enhance systemic tumor-specific immune responses.

In summary, these study results collectively support the potential of cryoablation combined with ICB therapy in the treatment of various cancers. This combined modality has not only demonstrated good tolerability and safety but also shown significant efficacy in enhancing anti-tumor immune responses. These findings provide important scientific evidence for the further clinical application of cryoablation and ICB therapy. However, more clinical research and long-term follow-up are needed to thoroughly understand the mechanisms of this combined treatment strategy, and to assess its long-term efficacy and applicability.

### 4.3 The potential advantages of cryoablation combined with immune checkpoint blockade

Firstly, ICB has been approved by the FDA for cancer treatment, and cryoablation has also been widely accepted in cancer therapy (105–115). Therefore, this combination treatment approach holds practical clinical value.

Secondly, cryoablation may enhance the sensitivity of tumor cells to ICB. The expression level of PD-L1 is often considered as an indicator for predicting the tumor’s response to ICB (116). Studies have shown that cryoablation may increase the expression levels of PD-L1 in tumor tissues and PD-1 on T cell surfaces, thereby enhancing the sensitivity of tumor cells to ICB. For example, Ock et al. found an upregulation of PD-L1 and PD-1 expression in head and neck squamous cell carcinoma (HNSCC) patients within one week after cryoablation treatment. Zhu C et al. (93) demonstrated in a renal cell carcinoma mouse model study that cryoablation can increase the expression of PD-L1 in tumor cells and PD-1 in CD8^+^ T cells.

Thirdly, tumor neoantigens released by cryoablation may help alleviate the problem of acquired resistance to ICBs (117, 118). During the use of ICB for tumor treatment, cancer cells may lose the most immunogenic mutations through intense immune selection pressure via cancer immune editing processes (119–121), or reduce mutations or expressions of genes associated with antigen presentation pathways, thereby reducing T cell recognition. This may result in primary or acquired resistance of tumors to PD-1 blockers (122, 123). Recent research has found that tumor tissues of NSCLC patients with acquired resistance to immune checkpoint blockade may have 7-18 presumed mutation-associated neoantigens missing (124, 125). Therefore, increasing the exposure of neoantigens may be a way to alleviate acquired resistance to ICB. Previous studies have shown that *in situ* tumor ablation techniques, such as radiofrequency and cryoablation, can lead to extensive tumor cell lysis, and the fragmented tumor cells may release more neoantigens, increasing the exposure of tumor neoantigens and reinducing endogenous immune responses (126, 127). The combination of ICB with localized cryoablation therapy may provide a valuable approach to enhance immune cell recognition of tumor neoantigens (117, 118). Therefore, in the face of the increasingly common occurrence of acquired resistance to immune checkpoint blockade, cryoablation may become a new combination therapy for ICB-resistant patients by increasing the release of neoantigens. In a case report of a 75-year-old female patient with advanced NSCLC (117), cryoablation successfully prevented the recurrence of lymph node metastasis in the aortic region, in a patient who had acquired resistance to ICB. This case suggests that cryoablation can provide a successful, safe, and feasible strategy to enhance the anti-tumor effect of ICB and may help overcome acquired resistance to ICB.

## 5 Conclusion

In summary, the combination of cryoablation and ICB therapy has two important considerations. On one hand, ICB can enhance the weak anti-tumor immune response induced by cryoablation. When ICB blocks the immune checkpoints that cause T cell exhaustion, the circulating cryoablation-induced antigens recognized by the immune system can bypass the tumor’s immune checkpoint escape mechanism, resulting in a potent anti-tumor T cell response. On the other hand, cryoablation may enhance the expression of PD-L1 on tumor tissues and PD-1 on CD8^+^ T cells, thereby increasing the sensitivity of tumor cells to ICB. Additionally, the release of a large number of tumor neoantigens by cryoablation may help alleviate the issue of acquired resistance to ICB.

The combination of cryoablation and ICB therapy is an effective strategy that outperforms monotherapy. This combination therapy has shown significant effects in enhancing the patient’s anti-tumor immune response and eliminating tumors, providing strong theoretical support for the clinical integration of cryoablation and ICB, bringing new hope for patients with advanced metastatic tumors.

## 6 Prospects

Currently, research on the combined treatment of cancer using cryoablation technology and ICB remains relatively limited. We need to develop various solid tumor models and employ techniques such as RNA sequencing, single-cell sequencing, flow cytometry, and multiplex immunohistochemistry. These methods will allow us to thoroughly analyze the intensity of the anti-tumor immune response elicited by the combined treatment, as well as changes in the tumor microenvironment. This will help us to more comprehensively and precisely assess the response of different types of tumors to the combination therapy and clearly define its potential mechanisms of action.

Furthermore, to validate the tolerability, safety, and efficacy of this combination therapy, extensive clinical studies are also necessary. These studies should cover key issues such as the optimal parameter settings for cryoablation (such as duration, number of freeze-thaw cycles, volume of the ablated tumor), the ideal dosage and administration method for ICB, as well as the sequence of cryoablation and ICB treatment, and the appropriate duration of combination therapy for different types of tumors. Given that the efficacy of the combination treatment may be influenced by factors such as tumor tissue type, individual patient differences, and variations between organs, even the same treatment protocol may yield different immunological effects in different patients. Therefore, in clinical application, it is crucial to optimize treatment parameters personalized to the specific situation of each tumor patient, in order to develop the most effective combination therapy plan.

As we explore the possibilities of future combination therapies, we might consider the depletion of Tregs as a potential approach, which could help enhance the efficacy of cryo-immunomodulation multimodal therapies. Tregs play a key role in suppressing the effector functions of CD8^+^ T cells and have long been considered a factor influencing the effectiveness of ICB therapies, as well as a potential target in treatment strategies (128). Interventions targeting Tregs can alter the immunosuppressive state within the tumor microenvironment, potentially eliciting a more effective anti-tumor immune response. This theoretically may enhance the efficacy of combined cryoablation and ICB therapy. For instance, cyclophosphamide has been proven to effectively reduce the number of Tregs in the tumor microenvironment and increase the presence of tumor-infiltrating T cells producing IFN-γ, thereby strengthening the anti-tumor immune response (129–131). Consequently, strategies to deplete Tregs might serve as an effective supplement to cryoablation combined with ICB therapy in the future. Future research could explore how to optimize the immunomodulatory effects of cryoablation and ICB combination therapy through pharmacological intervention in Treg cells, potentially offering a new perspective in cancer treatment. Although these forward-looking views provide new ideas for future combination therapies, their clinical application and long-term effects still need further exploration and validation in future scientific research.

## Author contributions

QL: Conceptualization, Writing – original draft. CZ: Writing – review & editing. XC: Writing – review & editing. ZH: Funding acquisition, Resources, Supervision, Writing – review & editing.
